# C5a/C5aR1 Pathway Is Critical for the Pathogenesis of Psoriasis

**DOI:** 10.3389/fimmu.2019.01866

**Published:** 2019-08-07

**Authors:** Quan-you Zheng, Shen-ju Liang, Feng Xu, Gui-qing Li, Na Luo, Shun Wu, You Li, Ming Tang, Yu Zhong, Jian Chen, Di Yang, Dao-dong Sun, Ke-qin Zhang, Gui-lian Xu

**Affiliations:** ^1^Department of Immunology, Army Medical University (Third Military Medical University), Chongqing, China; ^2^Department of Urology, 958th Hospital of PLA, Army Medical University (Third Military Medical University), Chongqing, China; ^3^Department of Rheumatism and Immunology, Daping Hospital, Army Medical University (Third Military Medical University), Chongqing, China; ^4^Department of Dermatology, Southwest Hospital, Army Medical University (Third Military Medical University), Chongqing, China; ^5^Department of Nephrology, Southwest Hospital, Army Medical University (Third Military Medical University), Chongqing, China

**Keywords:** complement system, C5a/C5aR1, psoriasis, inflammation, mouse models

## Abstract

Psoriasis is one of the most common chronic inflammatory skin diseases, affecting ~2% of the population. The lack of characterization of the pathogenesis of psoriasis has hindered efficient clinical treatment of the disease. In our study, we observed that expression of complement component 5a receptor 1(C5aR1) was significantly increased in skin lesions of both imiquimod (IMQ) and IL23-induced psoriatic mice and patients with psoriasis. C5aR1 deficiency or treatment with C5a receptor 1 antagonist (C5aR1a) in mice significantly attenuated psoriasis-like skin lesions and expression of inflammatory cytokines and chemokines. Moreover, C5aR1 deficiency significantly decreased IMQ-induced infiltration of plasmacytoid dendritic cells (pDCs), monocytes and neutrophils in psoriatic skin lesions and functions of pDCs, evidenced by the remarkable reduction in the IMQ-induced production of interferon-α (IFN-α) and tumor necrosis factor α (TNF-α), and FMS-like tyrosine kinase 3 ligand (FLT3L)-dependent pDCs differentiation. Accordingly, *in vitro* treatment with recombinant C5a accelerated pDCs migration and the differentiation of bone marrow cells into pDCs. Furthermore, biopsies of psoriatic patients showed a dramatic increase of C5aR1^+^ pDCs infiltration in psoriatic skin lesions, compared to healthy subjects. Our results provide direct evidence that C5a/C5aR1 signaling plays a critical role in the pathogenesis of psoriasis. Inhibition of C5a/C5aR1 pathway is expected to be beneficial in the treatment of patients with psoriasis.

## Introduction

Psoriasis is one of the most common chronic inflammatory skin diseases, affecting ~2% of the world's population (1). Psoriatic lesions are characterized by various histological features, including epidermal hyperplasia caused by excessive proliferation of keratinocytes, termed “acanthosis;” parakeratosis caused by impaired differentiation of keratinocytes; hypervascularity; and immune cell infiltration by neutrophils, dendritic cells, T cells, and macrophages (2, 3). Although the underlying mechanism is not fully understood, increasing evidence indicates that the innate immune system plays an essential role in the development of psoriasis (4–7).

The complement system, one of the most important arms of innate immunity, comprises a series of soluble and cell surface proteins, including plasma components, specific receptors, and diverse regulators. The complement system serves as the first line defense against the foreign pathogens by applying complement activation elements acting with antibodies, phagocytes, or through the formation of the terminal membrane attack complex (MAC) (8–10). Complement component 5a (C5a), a powerful, pro-inflammatory anaphylatoxin synthesized after cleavage of complement component C5, exerts its biological action by binding to its specific receptor C5aR1, also known as CD88, expressed on macrophages, neutrophils, monocytes, keratinocytes, and human plasmacytoid dendritic cells (pDCs) (11–13). A second receptor of C5a, C5-like receptor 2 (C5L2), was identified to be a negative regulator of C5a-dependent signaling (14).

Multiple reports indicated that excessive production of C5a leads to deleterious exaggeration of of the innate and adaptive immune responses, contributing to the development of several diseases such as allograft ischemia-reperfusion injury (IRI) and sepsis (15–17). Furthermore, high levels of cleaved C5a and C5a des-Arg were exhibited in skin lesions and psoriatic scales (18–21). However, the role of C5a/C5aR1 signaling in the development and progression of psoriasis remains unclear.

Although psoriasis has not been detected in any mammalian species other than humans, there exist several mouse models that mimic certain features of the human skin disease. Imiquimod (IMQ), a synthetic agonist for toll-like receptor 7 (TLR7), is a potent immune response activator and induces psoriasis-like skin inflammation (4). Significant increase in pDCs infiltration and abundant expression of type I interferon are critical hallmarks of IMQ-induced psoriasis in animal models (22, 23). However, the role of the C5a/C5aR1 signaling pathway in pDCs function in the pathogenesis of psoriasis is unknown.

In the present study, we explored the role of the C5a/C5aR1 complement pathway in the development of psoriasis and the potential relationship between C5aR1 signaling and inflammatory cells including pDCs, monocytes and neutrophils using IMQ-induced psoriasis in mice as the model. Our results suggest that C5a/C5aR1 signaling plays a critical role in the pathogenesis of psoriasis.

## Materials and Methods

### Mice and Treatment

C5aR1-deficient BALB/c mice were purchased from the Jackson Laboratory. Wild-type BALB/c mice were obtained from the animal institute of the Academy of Medical Science (Beijing, China). Animals were bred in a specific-pathogen-free facility (SPF), and female mice of 8–12 weeks were used for all experiments (Although both male and female BALB/c mice were susceptible for IMQ or IL23- induced psoriasis, the docile character of female mice is convenient to applicate IMQ evenly on the back skin of mice under non-anesthesia, we used female mice in the present study, but still with a limitation that it may not fully represent some of the behaviors of male mice since hormonal effects). Animal experiments were conducted according to the guidelines of the Institutional Animal Care and Use Committee of the Third Military Medical University.

Female mice of 8–12 weeks were treated with daily topical doses (62.5 mg) of commercially available IMQ cream (5%; Si-Chuan Med-Shine Pharmaceutical, H20030128, Chengdu, Sichuan, China). Treatment was applied to the shaved backs and ears of mice for the indicated periods of time. Mice within the control group were treated with Vaseline cream. C5aR1 peptide antagonist (24) (peptide sequence: Ac-Phe-[Orn-Pro-dCha-Trp-Arg]) (1 mg/kg, equal to 1.1 μM/kg; GL Biochem, Shanghai, China) was injected into mice via peritoneal cavity 2 h prior to IMQ application. Saline buffer (0.9%) was injected as a vehicle control.

### Intradermal Injection of Interleukin 23 (IL-23)

Mice were anesthetized and PBS (20 μL), either alone or containing recombinant mouse IL-23 (500 ng; Biolegend, San Diego, CA, USA), was injected intradermally into the ears, using a 30 gauge needle, for five consecutive days. Ear thickness was measured prior to injection at day 0, and thereafter, on days following cytokine injections, using a dial thickness gauge (Jiuliang, Shanghai, China). All tests were performed blinded.

### Measuring Skin Inflammation

To assess psoriatic lesion severity, an objective scoring system based onclinical Psoriasis Area and Severity Index (PASI) was used. Scaling, thickness, and erythema of the back skin were scored daily for severity on a scale from 0 to 4, where 4 represented maximum severity. Level of erythema was scored using a table of red taints, as previously described (22). Total modified PASI (mPASI) scores (0-12) were used as a measure of skin lesion severity before and after IMQ application.

### Human Blood and Skin Samples

Human blood samples were collected from psoriatic patients (*n* = 15) and healthy subjects (*n* = 15). Peripheral blood mononuclear cells (PBMCs) were separated by Ficoll density gradient centrifugation (TBD Science, Tian Jin, China). Skin paraffin sections were obtained from psoriatic patients after histological confirmation (*n* = 8) and biopsies sections were only collected from patients with no exposure to topical or systemic treatment for at least 2 weeks. Healthy control skin paraffin sections were obtained from individuals undergoing plastic surgery (*n* = 5). Patient studies were approved by the Institutional Ethical Board of the Army medical university (Third Military Medical University) and written informed consent was obtained from all subjects.

### Histopathological Analysis

Back or ear skin samples were collected from mice. Then, skin samples were either fixed in 4% paraformaldehyde and embedded in paraffin for hematoxylin and eosin (H&E) staining, or immersed in Tissue Tek (Leica Microsystem, Wetzlar, Germany), snap frozen in liquid nitrogen, and stored at −80°C. For immunohistochemistry assay, paraffin sections (4 μm) were incubated with rabbit anti-mouse/human C5aR1 polyclonal antibody (1:100, Abcam, Cambridge, UK) and rat anti-mouse Ki67 (1:100, SolA15, eBioscience, San Diego, CA, USA) overnight at 4°C and then incubated with horse radish peroxidase (HRP)-labeled anti-rabbit and anti-rat secondary antibodies, respectively (1:1000, Beyotime, Shanghai, China) for 60 min at room temperature. After washing in PBS, 3,3′-diaminobenzidine chromogen solution (Beyotime, Shanghai, China) was added for 2 min at room temperature. Slides were counterstained with hematoxylin and then examined via light microscope (Olympus, Tokyo, Japan). For immunofluorescence assay, cryosections were cut into 6 μm slices using a cryostat (Leica Microsystem, Wetzlar, Germany) and fixed in cold acetone for 15 min on ice (IF), slides were blocked with PBS containing 5% BSA for 60 min. Slides were incubated with antibodies for Alexa Fluor 488 labeled rat anti-mouse PDCA-1 (1:200, eBio927, eBioscience, San Diego, CA, USA) overnight at 4°C. Human skin samples paraffin sections (4 μm) were stained with rabbit anti-human/mouse C5aR1 polyclonal antibody (1:100, Abcam, Cambridge, UK) and mouse anti-human CD123 (1:100, 6H6, eBioscience, San Diego, CA, USA) at 4 °C overnight. Horse radish peroxidase (HRP) labeled secondary antibodies (1:1000, Beyotime Shanghai, China) or Dylight™ 488 donkey anti-mouse IgG and Cy3^TM^ goat anti-rabbit IgG (1:200, BioLegend, San Diego, CA, USA) were incubated for 60 min at room temperature. Sections were incubated with an antibody of the same isotype as a control. Slides were counterstained with hematoxylin and then examined via light microscope (Olympus, Tokyo, Japan) or slides were stained with Hoechst 33258 (5 μg/mL; Sigma-Aldrich, St. Louis, MO, USA) for nucleus staining and visualized via confocal laser microscope (Leica Microsystem, Wetzlar, Germany).

Ki67^+^, PDCA-1^+^ and C5aR1^+^CD123^+^ cells were quantified by counting the number of positively stained cells. 5–8 viewing fields randomly selected for each skin sections were examined and expressed as a number of cells per filed (0.04 mm^2^). The mentioned quantitative analysis were conducted in a bind fashion by 2 experienced persons.

### Quantitative Reverse Transcription-Polymerase Chain Reaction (qRT-PCR) Analysis

Total RNA was obtained from back or ear skin samples of mice, using TRIzol reagent (Biomed, Beijing, China). cDNA was synthesized via PrimeScript^TM^ RT reagent kit (Takara, Shiga, Japan). Real-time PCR was conducted with an MxPro3000P (Agilent StrataGene, America) and SYBR® Premix Ex Taq^TM^ reagent kit (Takara, Shiga, Japan). PCR mix (25 μL) included cDNA (2 μL) and primer pairs (0.5 μmol) for the target genes or GAPDH (listed in Table 1). Amplification was performed according to the manufacturer's protocol, and experiments were conducted in triplicate. Gene expression was quantified by ΔΔCt method (25). Samples were normalized to GAPDH.

**Table 1:** PCR primer sequences.

**Gene**	**Primer sequence**
C5aR1 Forward	5-ATGGACCCCATAGATAACAGCA-3
C5aR1 Reverse	5-GAGTAGATGATAAGGGCTGCAAC-3
PDCA-1 Forward	5-TGTTCGGGGTTACCTTAGTCA-3
PDCA-1 Reverse	5-GCAGGAGTTTGCCTGTGTCT-3
GAPDH Forward	5-ACCACAGTCCATGCCATCAC-3
GAPDH Reverse	5-TCCACCACCCTGTTGCTGTA-3
IFN-α Forward	5-CCAAGTGCTGCCGTCATTTTC-3
IFN-α Reverse	5-GGCTCGCAGGGATGATTTCAA-3
IFN-γ Forward	5-ATGAACGCTACACACTGCATC-3
IFN-γ Reverse	5-CCATCCTTTTGCCAGTTCCTC-3
Krt10 Forward	5-TTTGGTGGCGGTACTATGGAG-3
Krt10 Reverse	5-CTCTCGCTGGCTTGAGTTG-3
MIP-1α Forward	5-TTCTCTGTACCATGACACTCTGC-3
MIP-lα Reverse	5-CGTGGAATCTTCCGGCTGTAG-3
MIP-2 Forward	5-CCAACCACCAGGCTACAGG-3
MIP-2 Reverse	5-GCGTCACACTCAAGCTCTG-3
TNF-α Forward	5-TCTTCTCATTCCTGCTTGTGG-3
TNF-α Reverse	5-GGTCTGGGCCATAGAACTGA-3
IL-22 Forward	5-ATGAGTTTTTCCCTTATGGGGAC-3
IL-22 Reverse	5-GCTGGAAGTTGGACACCTCAA-3
IL-l7A Forward	5-TTTAACTCCCTTGGCGCAAAA-3
IL-l7A Reverse	5-CTTTCCCTCCGCATTGACAC-3
IL-23A Forward	5-ATGCTGGATTGCAGAGCAGTA-3
IL-23A Reverse	5-ACGGGGCACATTATTTTTAGTCT-3

### Bone Marrow pDCs Induction and Differentiation

Bone marrow (BM) pDCs were generated from C5aR1^+/+^ and C5aR1^−/−^ mice using a modified protocol previously described (26, 27). Briefly, BM cells were harvested from mouse femurs and tibias. BM cells (2 × 10^6^) from C5aR1^+/+^ and C5aR1^−/−^ mice were seeded in 6-well plates and cultured with RPMI 1640 medium containing 10% fetal calf serum, streptomycin (100 μg/mL), penicillin (100 U/mL), 2 μM glutamine, 50 μM β-mercaptoethanol, and recombinant mouse FLT3L (200 ng/mL; eBioscience, San Diego, CA, USA). Half culture medium was replaced every 5 days. In the another experiment, BM cells from C5aR1^+/+^ mice were simulated with FLT3L alone or the combination of FLT3L and recombinant mouse C5a (42 nM; Peprotech, Rocky Hill, NJ, USA), and medium group was used as the negative control. At day 10, detached cells were collected and analyzed the percentage of pDCs by FCM.

### ELISA

pDCs were isolated from mouse splenocytes with EasysepTM Mouse Plasmacytoid DC Isolation Kit (Stem cell Technologies, London, UK). The purity of pDCs was routinely at least 80% (Figure S9). Sorted pDCs from C5aR1^+/+^ and C5aR1^−/−^ mice were cultured (1 × 10^5^ cells) in a 96-well round-bottom plate for 24 h in the presence of IMQ (10 μg/mL) or resiquimod R848 (10 μg/mL; Sigma-Aldrich, St. Louis, MO, USA), and medium group was used as the negative control. The levels of interferon α (IFN-α) and tumor necrosis factor α (TNF-α) in culture supernatants were measured using mouse IFN-α and TNF-α ELISA kits (BioLegend, San Diego, CA, USA). C5a levels in culture supernatants of the medium group and in serum of naïve C5aR1^+/+^ and C5aR1^−/−^ mice were measured with commercial ELISA kit from USCN (SEA388Mu, Wuhan, China), according to the manufacturer's instruction.

### Flow Cytometry

The shaved back of C5aR1^+/+^ and C5aR1^−/−^ mice were topically applied with IMQ for indicated times or the ears of C5aR1^+/+^ mice were intradermally injected with IL-23 for 5 days. Cells were isolated from psoriatic skin lesion samples. Briefly, skin samples were minced and digested in collagenase type IV (2 mg/mL; Sigma-Aldrich, St. Louis, MO, USA) and DNase I (200 U/mL; Sigma-Aldrich, St. Louis, MO, USA) for 60 min at 37°C. Cells at 1 × 10^6^ cells/ml were incubated with Fc blocking for 20 min on ice and then stained with PE-labeled rat anti-mouse C5aR1 (1 μl/test, 20/70, BioLegend, San Diego, CA, USA), PerCP-Cy5.5- labeled rat anti-mouse Gr-1 (1 μl/test, RB6-8C5, Biolegend, San Diego, CA, USA), PE-Cy7-labeled rat anti- mouse CD11b (1 μl/test, CBRM1/5, BioLegend, San Diego, CA, USA), or FITC-labeled rat anti-mouse PDCA-1 (1 μl/test, eBio927, eBioscience, San Diego, CA, USA) antibodies for 25 min on ice in the dark. For bone marrow derived pDCs, cells were stained with FITC-labeled rat anti-mouse PDCA-1. For pDCs purified from mouse splenocytes, the purity was measured by FITC-labeled rat anti-mouse PDCA-1 and PE-labeled rat anti-mouse B220 (1 μl/test, RA3-6B2, Biolegend, San Diego, CA, USA). Sorted pDCs from C5aR1^+/+^ mice were cultured (1 × 10^5^ cells) in a 96-well round-bottom plate for 24 h in the presence of IMQ, and was stained with PE-labeled rat anti-mouse C5aR1 antibody. For human PBMCs from psoriatic patients and healthy subjects, cells were stained with PE-labeled mouse anti-human C5aR1 (1 μl/test, S5/1, BioLegend, San Diego, CA, USA) and FITC-labeled rat anti-human CD123 (1 μl/test, 6H6, eBioscience, San Diego, CA, USA). Corresponding rat or mouse isotype IgG was used as the negative control. Stained cells were assessed by flow cytometry (BD FACS Canto II, Franklin Lakes, NJ, USA) and data were analyzed using Flow Jo software (Tree Star, Ashland, OR, USA). The acquired cells number for each sample keeps in the range of 2 × 10^5^~1 × 10^6^ depending on different situations.

### Chemotaxis Assay

Chemotaxis migration assays were performed using 24-well transwell plates (5 μm pore size; Corning, NY 14831, USA). pDCs were purified from mouse splenocytes and seeded (1 × 10^6^ cells) in the upper chamber and chemoattractant medium (600 μL) containing C5a (42 nM; PeproTech, Rocky Hill, NJ, USA) was added to the lower chamber. Transwell plates were incubated for 90 min at 37°C in the presence of 5% CO_2_. Migrated cells in the lower chamber were collected and counted by FCM. Experiments were performed in triplicate and results were calculated as mean values of three experiments.

### Statistical Analysis

Results were presented as mean ± standard error of the mean (SEM). GraphPad Prism 5.0 software (GraphPad Software, Inc., La Jolla, CA) was used for statistical analysis. The student's *t*-test or two-way ANOVA were used to compare samples, and *p* < 0.05 was considered statistically significant.

## Results

### Increased C5aR1 Expression in Psoriatic Mouse Skin Lesions

**Figure 1:**
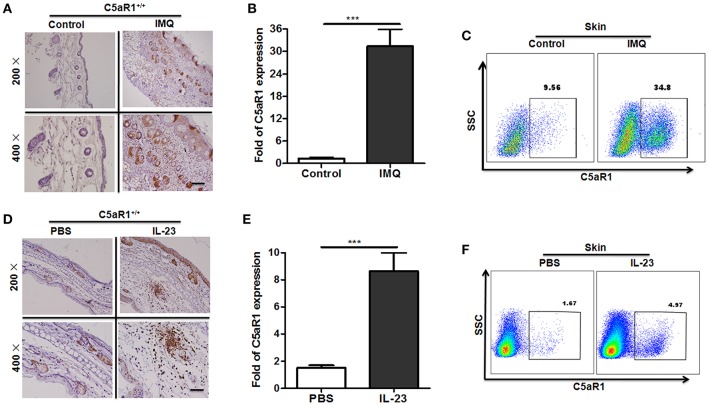
Increased C5aR1 expression in psoriatic skin lesions in mice. To induce a psoriatic animal model, IMQ was topically applied to the shaved back of wild-type (C5aR1^+/+^) mice for 6 days **(A–C)**, or the ears of wild-type (C5aR1^+/+^) mice were intradermally injected with IL-23 for 5 days **(D–F)**. Samples were collected and tested for C5aR1 expression (*n* = 5/group). **(A,D)** C5aR1 expression in local skin tissues was detected by IHC. **(B,E)** C5aR1 mRNA levels in skin tissues were analyzed. **(C,F)** C5aR1 expression in infiltrated cells from skin lesions was measured by FCM. Values are presented as mean ± SEM (*n* = 5/group). Gating strategy for **(C,F)**, isotype control for **(A,D)**, and negative control of C5aR1 expression was shown in Figure S1. Data are representative of two independent experiments. ^***^p < 0.001.

**Figure 2:**
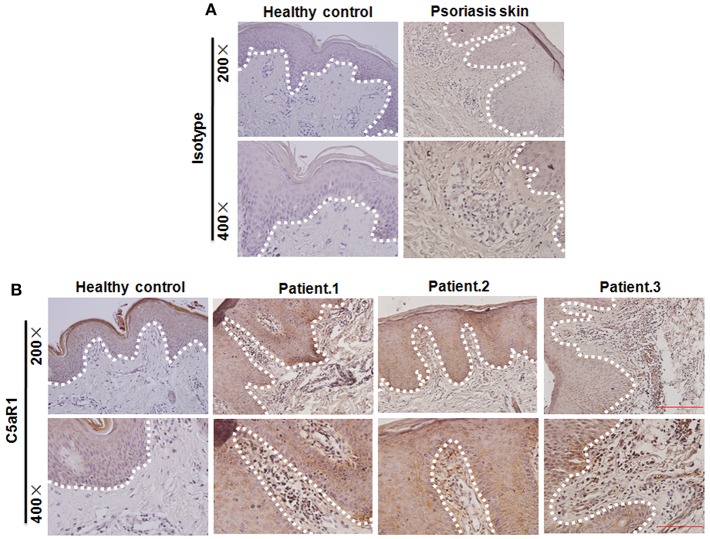
Increased C5aR1 expression in psoriatic skin lesions in patients with psoriasis. **(A)** Isotype control and **(B)** C5aR1 expression by Immunohistochemistry. Healthy volunteer was used as healthy control (*n* = 8 for psoriasis patient; *n* = 5 for healthy volunteer). White dotted line: the location of the basal membrane.

Abundant levels of C5a des-Arg were extracted from psoriatic scales (19, 21). As shown in Figures 1A–C, C5aR1 expression was significantly increased in the local skin lesions tissues and in cells isolated from skin lesions in IMQ-induced psoriatic mice, compared to the control mice, according to immunohistochemistry (IHC), qRT-PCR, and FCM analyses. Moreover, C5aR1 expression of IMQ-treated back skin tissues in mice was predominantly on keratinocytes including the epidermis and hair follicles as wells as skin tissues infiltrating cells. Similar results were observed when mice were treated with recombinant IL-23 in ears (Figures 1D–F), which is another well-defined mouse model of psoriatic skin inflammation (28, 29). Consistent with the results in psoriatic mice, C5aR expression in psoriatic skin tissues from patients with psoriasis was also significantly increased, compared with that from healthy controls (Figure 2).

All these results suggest that C5aR1 signaling pathway is closely related to the pathogenesis of psoriasis.

### C5aR1 Deficiency Reduces Severity of Psoriasis

**Figure 3:**
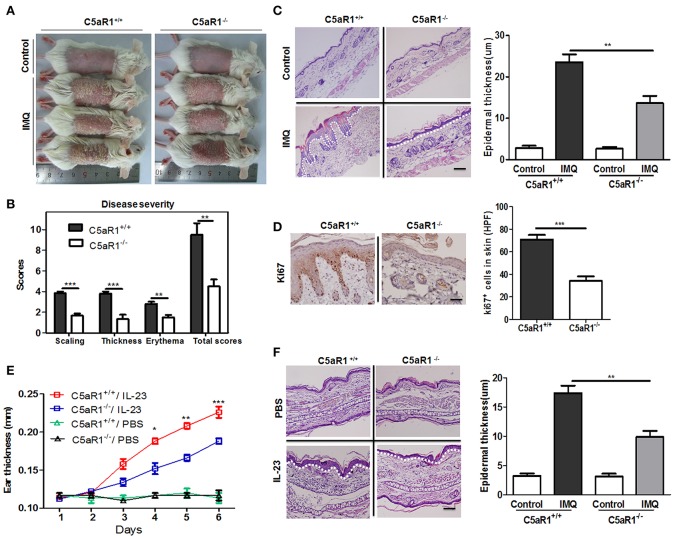
C5aR1 deficiency reduced psoriatic skin inflammation. Wild-type (C5aR1^+/+^) and C5aR1-deficient (C5aR1^−/−^) mice were treated with IMQ for six consecutive days or intradermally injected with IL-23 for 5 days, skin samples were collected 24 h after the final IMQ application **(A–D)** or IL-23 injection **(E,F)**. **(A)** Macroscopic phenotype of psoriatic lesions in C5aR1^+/+^ and C5aR1^−/−^ mice treated with IMQ or Vaseline cream (control). **(B)** Disease severity was scored by a modified clinical Psoriasis Area and Severity Index (mPASI). The cumulative mPASI scores after 6 days treatment with IMQ were presented. **(C)** H&E staining of mice skin lesion sections. Epidermal thickness of the skin was measured. Scale bar = 20 μm. **(D)** IHC staining for Ki67 in skin lesion sections. Right: Ki67-positive cells quantification. **(E)** Ear thickness was measured following IL-23 injection. **(F)** H&E staining of ear skin lesion sections. Epidermal thickness of the ears was measured. White dotted line: the location of the basal membrane. Scale bar = 5 μm. Values are presented as mean ± SEM. Data are representative of three independent experiments and each group consisted of more than five mice. ^*^p < 0.05; ^**^p < 0.01; ^***^p < 0.001.

To determine the importance of the C5aR1 signaling pathway in psoriasis, we used C5aR1-deficient mice. As shown in Figures 3A,B, topical application of IMQ on the shaved back of wild-type C5aR1^+/+^ mice for six consecutive days induced psoriatic lesions, including erythema, scales, and crust formation, as previously reported (22). Interestingly, IMQ-induced psoriasis-like skin inflammation and total mPASI scores of C5aR1^−/−^ mice were significantly less than those of C5aR1^+/+^ mice (Figures 3A,B). Furthermore, we observed a significant decrease in the epidermal thicknesses (Figure 3C) and the occurrence of parakeratosis (Figure S2) in C5aR1^−/−^ mice, compared to C5aR1^+/+^ mice. Expression levels of Ki67, a marker for cells proliferation, was significantly reduced in the local skin tissues of IMQ-treated C5aR1^−/−^ mice (Figure 3D). Similar attenuation effects in psoriatic development were observed in the ear skin samples of IMQ-treated and IL-23-treated C5aR1^−/−^ mice (Figure S3 and Figures 3E,F).

**Figure 4:**
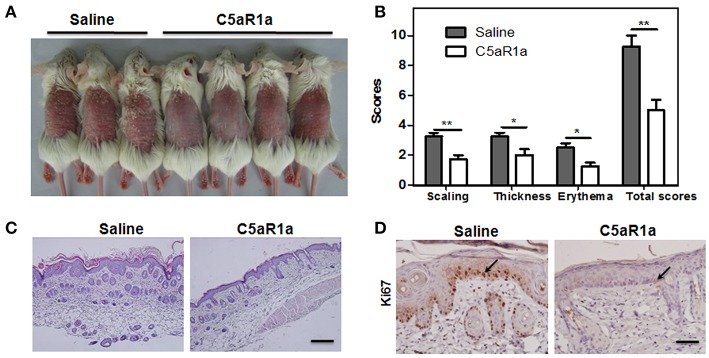
Blockage of C5aR1 signaling with C5a receptor antagonist (C5aR1a) attenuated IMQ-induced psoriatic skin inflammation. C5aR1^+/+^ mice were pre-treated with C5aRa (1 mg/kg) or 0.9% saline for 2 h prior to treatment with IMQ. After IMQ treatment for six consecutive days, digital pictures were obtained and skin samples were collected. **(A)** Phenotype of IMQ-induced psoriatic skin lesions. The left three mice were pre-treated with saline, while the right four mice were pre-treated with C5aRa. **(B)** Disease severity was scored as described (*n* = 5–6/group). **(C)** H&E staining of psoriatic skin lesions. Scale bar = 20 μm. **(D)** IHC staining for Ki67 in psoriatic skin lesions. Scale bar = 5 μm. Arrows indicate positively stained cells. Data are representative of at least three independent experiments and each group consisted of more than five mice. ^*^p < 0.05; ^**^p < 0.01.

To validate the function of C5aR1 signaling in the pathogenesis of psoriasis, we inhibited the C5aR1 signaling pathway using a C5aR1 peptide antagonist (C5aR1a). As shown in Figure 4, C5aR1a-treated mice exhibited a significant attenuation in IMQ-induced psoriasiform skin inflammation (Figure 4A), total mPASI scores (Figure 4B), epidermal thicknesses (Figure 4C), and keratinocyte proliferation (Figure 4D).

These results clearly indicate that C5a/C5aR1 signaling is essential for the development of psoriasis.

### C5aR1 Deficiency Attenuates Inflammatory Cytokines and Chemokines Expression in Psoriatic Skin Lesions

**Figure 5:**
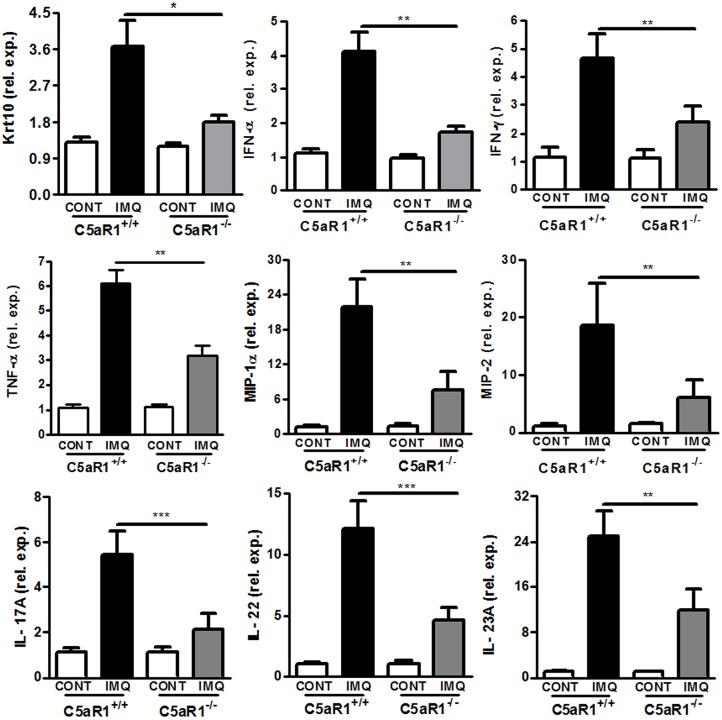
C5aR1 deficiency attenuated inflammatory cytokines and chemokines expression of psoriatic skin lesions. IMQ was topically applied to the shaved back of C5aR1^+/+^ and C5aR1^−/−^ mice for six consecutive days, and back skin samples were obtained on day 7. Expression levels of the indicated genes in psoriatic skin lesions were measured by qRT-PCR (*n* = 5–6/group). Values are presented as mean ± SEM and data were obtained from at least three independent experiments. ^*^p < 0.05; ^**^p < 0.01, ^***^p < 0.001.

Increased expression of inflammatory cytokines and chemokines plays a critical role in the progression of IMQ-induced skin inflammation (30–34). The expression levels of keratinocyte-specific factors and inflammatory cytokines were analyzed by qRT-PCR. Compared to C5aR1^+/+^ mice, skin tissues of C5aR1^−/−^ mice exhibited a significant reduction in mRNA levels of keratin 10 (Krt10), TNF-α, IFN-α/γ, IL-17A, IL-22, IL-23A, and the chemokines of T cells and neutrophils, macrophage inflammatory protein 1α (MIP-1α) and macrophage inflammatory protein 2 (MIP-2) (Figure 5). Similar results were observed in mice with C5aR1 signaling blockage (Figure S4).

Collectively, these results demonstrate that C5aR1 deficiency impairs production of inflammatory cytokines and chemokines in IMQ-induced skin lesions.

### C5aR1 Is Critical for pDCs Infiltration in Psoriatic Skin Lesions

**Figure 6:**
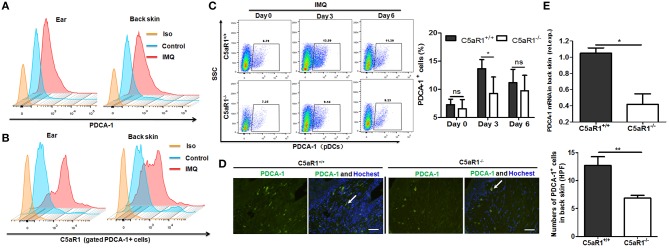
C5aR1 deficiency reduced pDCs infiltration in psoriatic lesions. C5aR1^+/+^ and C5aR1^−/−^ mice were treated with IMQ (Vaseline cream treatment as a control) on the shaved ear or back skin for indicated days, and then skin samples were obtained and infiltrated cells of skin lesions were isolated. **(A)** Frequencies of pDCs (PDCA-1^+^ cells) in the infiltrated cells of psoriatic ear and back skin lesions and **(B)** C5aR1 expression on these pDCs in C5aR1^+/+^ mice following 3 days IMQ application. **(C)** Frequencies of pDCs (PDCA-1^+^) in IMQ-treated back skin of C5aR1^+/+^ and C5aR1^−/−^ mice were compared at indicated times. Percentages of PDCA-1^+^ pDCs is shown (*n* = 5–6). **(D)** Infiltration of pDCs (PDCA-1^+^) on day 3 in IMQ-treated back skin tissues of C5aR1^+/+^ and C5aR1^−/−^ mice was measured by immunofluorescence. Arrows indicate positively stained cells. Scale bar = 5 μm. **(E)** PDCA-1 expression in IMQ-treated back skin tissues of C5aR1^+/+^ and C5aR1^−/−^ mice was analyzed by qRT-PCR following 3 days IMQ treatment. (*n* = 5–6/group). Gating strategy is shown in Figure S5. Values are presented as mean ± SEM and data were obtained from three independent experiments. ^*^p < 0.05; ^**^p < 0.01.

It has been reported that IMQ induces rapid pDCs accumulation and pDCs play a crucial role in the initiation of psoriatic lesions (23, 35, 36). Consistent with previous reports, we demonstrated that IMQ treatment significantly increased infiltration of pDCs (plasmacytoid dendritic cell antigen-1 positive, PDCA-1^+^ cells) in ear and back skin lesions (Figure 6A). Similar results were observed in monocytes and neutrophils (Figure S6). Moreover, C5aR1 expression was clearly enhanced in these infiltrated skin cells (Figure 6B and Figure S7). Accordingly, C5aR1 deficiency significantly reduced these inflammatory cells accumulation and local infiltration in IMQ-treated back skin lesions at day 3 or 6 (Figures 6C–E and Figure S6).

**Figure 7:**
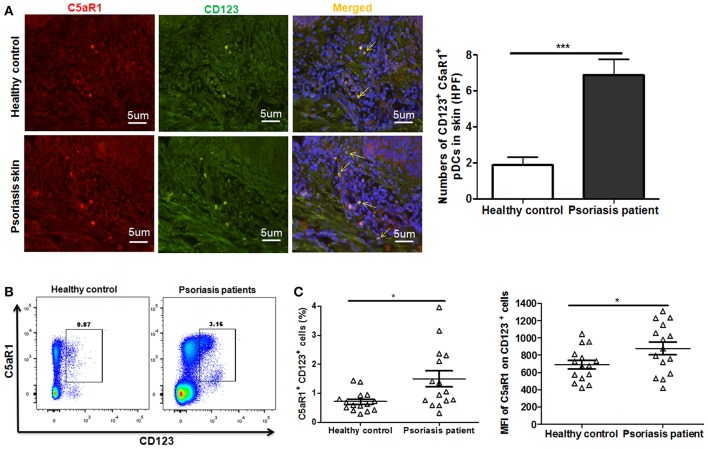
Increased C5aR1 expression on pDCs from psoriasis patients. Skin tissues and PBMCs of psoriatic patients and healthy individuals were collected. **(A)** Immunofluorescence staining for C5aR1 expression (red) on pDCs (stained with anti-CD123) (green) in skin tissues. Nuclei (blue) were stained with Hoechst 33258. Arrows indicate cells positively stained for both C5aR1 and CD123. Scale bar = 5 μm. Right: Quantification of C5aR1^+^ CD123^+^ cells is shown. **(B)** C5aR1 expression on pDCs (CD123^+^ cells) of PBMCs from psoriatic patients and healthy individuals (*n* = 15/group). Representative FCM analyses of C5aR1^+^CD123^+^ cells are illustrated. Gating strategy is shown in Figure S8. **(C)** Percentage of C5aR1^+^CD123^+^ cells and MFI of C5aR1 expression on pDCs (CD123^+^ cells). Values are presented as mean ± SEM and data were obtained from at least two independent experiments. ^*^p < 0.05; ^***^p < 0.001.

Similar results were observed in patients with psoriasis, as the frequency of C5aR1^+^ pDCs (CD123^+^ cells) substantially increased in both local skin tissues (Figure 7A) and PBMCs (Figures 7B,C), compared to healthy individuals.

All these results indicating that C5a/C5aR1 signaling pathway is essential for early pDCs accumulation in local skin during the development of psoriasis.

### C5aR1 Signaling Pathway Potentiates pDCs Function and Differentiation

**Figure 8:**
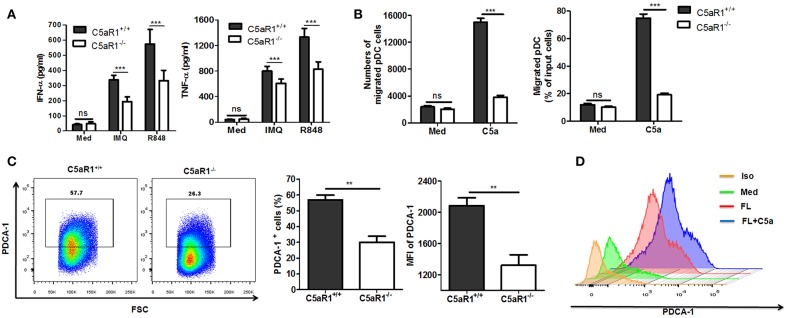
C5aR1 deficiency impaired pDCs function and differentiation *in vitro*. **(A,B)** pDCs were isolated from splenocytes of C5aR1^+/+^ and C5aR1^−/−^ mice. Culture medium was used as a control. **(A)** Purified pDCs (1 × 10^6^/ml) were stimulated with IMQ or R848 (10 μg/mL) *in vitro* for 24 hr. The levels of IFN-α and TNF-α in the culture supernatants were measured by ELISA. **(B)** pDCs (1 × 10^6^/ml) in transwell plates were exposed to C5a (42 nM) for 90 min. Cells that migrated to the lower chamber were measured. The purity of isolated pDCs and IMQ-induced C5aR1 expression of isolated pDCs are shown in Figure S9. C5a levels in cultural supernatant of purified pDCs and in serum of naïve C5aR1^+/+^ or C5aR1^−/−^ mice are shown in Figure S10. **(C)** BM cells from C5aR1^+/+^ and C5aR1^−/−^ mice were stimulated with recombinant mouse FLT3L (200 ng/ml for 10 days and the frequency of pDCs was measured by FCM. Left, a representative FCM graph. Right, average percentages and MFIs of PDCA-1^+^ cells. **(D)** BM cells from C5aR1^+/+^ mice were stimulated with recombinant mouse FLT3L (200 ng/mL) alone or with C5a (42 nM) for 10 days, culture medium was used as a control. The frequency of pDCs was analyzed by FCM. Values are presented as mean ± SEM and data were obtained from at least three independent experiments. ^**^p < 0.01; ^***^p < 0.001.

To further investigate the role of C5aR1 signaling in pDCs functions, we isolated pDCs from splenocytes of C5aR1^+/+^ and C5aR1^−/−^ mice. It was found that C5aR1 deficiency led to a significant decrease in the IMQ and R848-induced IFN-α and TNF-α secretion (Figure 8A) and in the chemoattractive capacity of pDCs, as assessed by transwell migration assay (Figure 8B). FLT3L has been reported to induce pDCs differentiation from bone marrow cells (BMs) (26, 27). To explore the effect of C5aR1 on pDCs differentiation, BMs from C5aR1^+/+^ and C5aR1^−/−^ were primed with FLT3L for 10 days, and then the percentage of pDCs was determined. It was showed that the proportion of pDCs was dramatically lower in FLT3L-stimulated BMs derived from C5aR1^−/−^ mice than those derived from C5aR1^+/+^ mice (Figure 8C). In accordance, treatment of BMs with recombinant mouse C5a further increased FLT3L-dependent differentiation of pDCs *in vitro* (Figure 8D).

Altogether, our data demonstrate the vital role of C5a/C5aR1 signaling in pDCs differentiation and functions.

## Discussion

Activation of the complement system was reported during the development of psoriasis and skin lesions exhibited high levels of activated C5a fragments (18–21). In agreement with these findings, our results showed that C5aR1 expression was clearly increased in keratinocytes (epidermis and hair follicles) as well as skin infiltrating cells from both psoriatic mice and patients with psoriasis. However, the role and underlying mechanisms of the complement C5a/C5aR1 signaling pathway in the pathogenesis of psoriasis remain unclear. In the present study, we reported that C5aR1 deficiency or inhibition with C5aR1a significantly reduced psoriasis-like skin inflammation, validating the importance of the C5a/C5aR1 pathway in IMQ-induced mouse psoriatic skin lesions. Furthermore, C5a/C5aR1 signaling potentiated pDCs, monocytes and neutrophils recruitment, and pDCs differentiation and functions, which suggest a possible mechanistic contribution of C5a/C5aR1 signaling in the pathogenesis of psoriasis, that requires further investigation in the future.

Multiple studies have demonstrated that IMQ-induced expression of pro-inflammatory cytokines and chemokines, specifically TNF-α, IFN-α/γ,IL-17A, IL-22, IL-23A, MIP-1α, and MIP-2, is essential for the development of psoriatic skin lesions (2, 3, 30). Our results provided evidence that C5a/C5aR1 signaling mediated development of psoriatic skin lesions, as C5aR1-deficient mice exhibited impaired expression of these genes.

Psoriasis is a chronic skin disease mediated by IFN-α- driven T cells, characterized by local accumulation of pDCs (23). pDCs are considered to be key in the development of psoriasis because of the significant production IFN-α (23). Moreover, it was previously demonstrated that TLR7 expression of pDCs was targeted by IMQ, representing an important upstream event that initiates the psoriatic skin inflammation (35, 36). However, the mechanism of regulation of pDC accumulation in psoriatic lesions is currently unknown. In our study, we showed that C5aR1 deficiency significantly reduced IMQ-induced pDCs infiltration, implicating the C5aR1 signaling pathway in pDCs infiltration in psoriasiform inflammation. Consistent with a previous study (13), we observed significantly increased C5aR1 expression in pDCs derived from PBMCs of psoriatic patients. There was a significant reduction in the production of IFN-α and TNF-α, a cytokine critical for the initiation of psoriasis, in cells treated with IMQ and R848. In addition, the chemo-attractive capacity of pDCs derived from C5aR1-deficient mice was significantly decreased. Altogether, we demonstrate that the C5a/C5aR1 pathway is critical for pDCs functions during the development of psoriasis in mice.

FLT3Lhas been shown to be an essential physiological factor for pDCs differentiation (26, 27). In the present study, C5aR1 deficiency clearly impaired FLT3L-dependent differentiation of pDCs from BMs, whereas exogenous recombinant C5a enhanced pDCs differentiation. These results suggest an essential role for C5aR1 signaling in FLT3L-dependent pDCs development. However, additional studies are necessary to investigate the underlying mechanisms by which C5a/C5aR1 signaling potentiates pDCs differentiation.

In conclusion, our study defined and characterized the role of C5a/C5aR1 signaling in the pathogenesis of IMQ-induced psoriatic lesions. Moreover, we demonstrated for the first time the dependency of C5a/C5aR1 signaling in pDCs differentiation and function, specifically in IMQ-induced psoriatic skin inflammation. Therefore, targeting C5a/C5aR1 interaction may prove to be a novel therapeutic approach for psoriasis.

## Data Availability

The raw data supporting the conclusions of this manuscript will be made available by the authors, without undue reservation, to any qualified researcher.

## Author Contributions

QZ and GX designed the study and prepared the manuscript. QZ, FX, SW, YL, MT, and GL carried out the experiments. NL and SL collected the specimens from patients and healthy donors. QZ, GX, JC, DY, DS, YZ, and KZ analyzed data. All authors made substantial contributions toward drafting the manuscript, reviewing the final manuscript for intellectual content, and authorizing the submission. That written informed consent was obtained from the participants of this study.

### Conflict of Interest Statement

The authors declare that the research was conducted in the absence of any commercial or financial relationships that could be construed as a potential conflict of interest.

## Supplementary Material

Figure S1 Isotype control and gating strategy of C5aR1 staining. After IMQ or IL-23 treatment, lesions skin sample of mice was collected and skin cells were isolated. **(A)** Skin single cell suspensions were stained with isotype IgG or anti-mouse C5aR1. The gating strategy is shown. **(B)** Skin paraffin sections were stained with rabbit IgG isotype antibody. **(C)** Skin paraffin sections from IMQ-treated C5aR1^−/−^ were stained with rabbit isotype IgG or rabbit anti-mouse C5aR1 antibody.

Figure S2 C5aR1 deficiency attenuated the parakeratosis of psoriatic skin in mice. The shaved back skin of C5aR1^+/+^ and C5aR1^−/−^ mice were topically applied with IMQ for 6 days. H&E staining of mice skin sections and one representative image analysis for parakeratosis are shown. White dotted line: the location of the basal membrane. The length of parakeratosis was measured per high-power field (HPF, 200×) from 5 to 6 mice per group. Values are presented as mean ± SEM and data were obtained from at least two independent experiments. ^**^*p* < 0.01.

Figure S3 C5aR1 deficiency alleviated IMQ-induced psoriatic skin inflammation in ears. C5aR1^+/+^ and C5aR1^−/−^ ears were topically treated with IMQ (Vaseline cream treatment as a control) for six consecutive days (*n* = 5–6 per group). Ear skin tissues were collected. **(A)** Macroscopic phenotype of psoriasiform lesions in C5aR1^+/+^ and C5aR1^−/−^ mice. **(B)** H&E staining of ear skin sections. Data represent three independent experiments.

Figure S4 C5aR1 antagonist treatment led to a reduction in the levels of inflammatory response genes in psoriatic skin lesions. Mouse C5aR1 antagonist (1 mg/kg) or 0.9% saline was injected via intraperitoneal cavity 2 h prior to IMQ application in C5aR1^+/+^ wild type mice. After six consecutive days of IMQ application, skin samples were collected and expression of inflammatory response genes were analyzed by qRT-PCR (*n* = 4–8). Values are presented as mean ± SEM and data were obtained from at least three independent experiments. ^*^*p* < 0.05; ^**^*p* < 0.01.

Figure S5 Gating strategy of pDCs staining. After IMQ treatment for indicated days, whole-skin cells were isolated from psoriatic skin lesions samples and stained with isotype IgG or anti-mouse PDCA-1 antibody.

Figure S6 C5aR1 deficiency led to decrease in neutrophils and monocytes infiltration of psoriatic skin lesions in mice. C5aR1^+/+^ and C5aR1^−/−^ mice were topically treated with IMQ for indicated days. Back skin samples were obtained. Infiltrated cells of skin lesions were isolated and the percentage of neutrophils and monocytes in the infiltrated cells was measured by FCM. **(A)** Gating strategy. **(B)** Monocytes (CD11b^+^ cells). **(C)** Neutrophils (Gr-1^+^ cells). (*n* = 5–6/group). Data were obtained from at least two independent experiments. ns, no significant, ^*^*p* < 0.05; ^**^*p* < 0.01.

Figure S7 Increased C5aR1 expression in infiltrated skin cells in psoriatic skin lesions in mice. To induce a psoriatic animal model, IMQ was topically applied to the shaved back of wild-type (C5aR1^+/+^) mice for six days. Samples were collected and cells were isolated from the skin lesions. **(A)** Gating strategy. **(B)** C5aR1 expression on pDCs (PDCA-1^+^), monocytes (CD11b^+^) and neutrophils (Gr-1^+^) was assayed by FCM. (*n* = 5/group). ^**^ *p* < 0.01.

Figure S8 Gating strategy of CD123 and C5aR1 staining in human PBMCs. Human PBMCs were prepared as described in methods (*n* = 15/group), and then stained with isotype IgG or anti-human CD123 and C5aR1 antibodies, followed by flow cytometric analysis.

Figure S9 The purity of isolated pDCs and IMQ-induced C5aR1 expression of isolated pDCs. pDCs were isolated from splenocytes of C5aR1^+/+^ mice. **(A)** The purity of isolated pDCs (PDCA-1 positive cells) was measured by FCM. **(B)** C5aR1 expression on pDCs after IMQ stimulation for 24 h was measured by FCM.

Figure S10 C5a levels in cultural supernatant of purified pDCs and in serum of naïve C5aR1^+/+^ or C5aR1^−/−^ mice. **(A)** pDCs were isolated from splenocytes of C5aR1^+/+^ or C5aR1^−/−^ mice and cultured in medium for 24 h. C5a levels in cell-free culture supernatant was measured by ELISA (*n* = 6/group). **(B)** C5a levels in serum from naïve C5aR1^+/+^ and C5aR1^−/−^ mice measured by ELISA (*n* = 5/group). Values are presented as mean ± SEM and data were obtained from at least two independent experiments.

## References

[1] Boehncke WH Schon MP. Psoriasis. Lancet. (2015) 386:P983–94. 10.1016/S0140-6736(14)61909-7 26025581

[2] Lowes MA Suarez-Farinas M Krueger JG. Immunology of psoriasis. Ann Rev Immunol. (2014) 32:227–55. 10.1146/annurev-immunol-032713-120225 24655295 PMC4229247

[3] Lowes MA Bowcock AM Krueger JG. Pathogenesis and therapy of psoriasis. Nature. (2007) 445:866–73. 10.1038/nature05663 17314973

[4] Wagner EF Schonthaler HB Guinea-Viniegra J Tschachler E. Psoriasis: what we have learned from mouse models. Nat Rev Rheumatol. (2010) 6:0704–14. 10.1038/nrrheum.2010.157 20877306

[5] Kondelkova K Krejsek J Borska L Fiala Z Hamakova K Ettler K. Membrane and soluble Toll-like receptor 2 in patients with psoriasis treated by Goeckerman therapy. Int J Dermatol. (2014) 53:e512–7. 10.1111/ijd.12381 25266302

[6] Kakeda M Schlapbach C Danelon G Tang MM Cecchinato V Yawalkar N. Innate immune cells express IL-17A/F in acute generalized exanthematous pustulosis and generalized pustular psoriasis. Arch Dermatol Res. (2014) 306:933–8. 10.1007/s00403-014-1488-0 25030504

[7] Nickoloff BJ Skin innate immune system in psoriasis: friend or foe? J Clin Invest. (1999) 104:1161–4. 10.1172/JCI8633 10545511 PMC409832

[8] Walport MJ. Complement. First of two parts. N Engl J Med. (2001) 344:1058–66. 10.1056/NEJM200104053441406 11287977

[9] Carroll MC. The complement system in regulation of adaptive immunity. Nat Immunol. (2004) 5:981–6. 10.1038/ni1113 15454921

[10] Ricklin D Hajishengallis G Yang K Lambris JD. Complement: a key system for immune surveillance and homeostasis. Nat Immunol. (2010) 11:785–97. 10.1038/ni.1923 20720586 PMC2924908

[11] Fayyazi A Sandau R Duong LQ Gotze O Radzun HJ Schweyer S C5a receptor and interleukin-6 are expressed in tissue macrophages and stimulated keratinocytes but not in pulmonary and intestinal epithelial cells. Am J Pathol. (1999) 154:495–501. 10.1016/S0002-9440(10)65295-9 10027407 PMC1849999

[12] Peng Q Li K Sacks SH Zhou W. The role of anaphylatoxins C3a and C5a in regulating innate and adaptive immune responses. Inflamm Allergy Drug Targets. (2009) 8:236–46. 10.2174/187152809788681038 19601884

[13] Gutzmer R Kother B Zwirner J Dijkstra D Purwar R Wittmann M. Human plasmacytoid dendritic cells express receptors for anaphylatoxins C3a and C5a and are chemoattracted to C3a and C5a. J Inves Dermatol. (2006) 126:2422–9. 10.1038/sj.jid.5700416 16778800

[14] Chen NJ Mirtsos C Suh D Lu YC Lin WJ McKerlie C. C5L2 is critical for the biological activities of the anaphylatoxins C5a and C3a. Nature. (2007) 446:203–7. 10.1038/nature05559 17322907

[15] Peng Q Li K Smyth LA Xing G Wang N Meader L. C3a and C5a promote renal ischemia-reperfusion injury. J Am Soc Nephrol. (2012) 23:1474–85. 10.1681/ASN.2011111072 22797180 PMC3431410

[16] Guo RF Ward PA. Role of C5a in inflammatory responses. Ann Rev Immunol. (2005) 23:821–52. 10.1146/annurev.immunol.23.021704.115835 15771587

[17] Ward PA. The harmful role of C5a on innate immunity in sepsis. J Innate Immun. (2010) 2:439–45. 10.1159/000317194 20588003 PMC2968761

[18] Kapp A Wokalek H Schopf E. Involvement of complement in psoriasis and atopic dermatitis–measurement of C3a and C5a, C3, C4 and C1 inactivator. Arch Dermatol Res. (1985) 277:359–61. 10.1007/BF00509233 3875319

[19] Ohkohchi K Takematsu H Tagami H. Increased C5a anaphylatoxin in the sera of psoriatic patients and patients with inflammatory dermatoses. J Dermatol. (1986) 13:266–9. 10.1111/j.1346-8138.1986.tb02939.x 3540054

[20] Takematsu H Ohkohchi K Tagami H. Demonstration of anaphylatoxins C3a, C4a and C5a in the scales of psoriasis and inflammatory pustular dermatoses. Br J Dermatol. (1986) 114:1–6. 10.1111/j.1365-2133.1986.tb02773.x 3484631

[21] Bergh K Iversen OJ Lysvand H. Surprisingly high levels of anaphylatoxin C5a des Arg are extractable from psoriatic scales. Arch Dermatol Res. (1993) 285:131–4. 10.1007/BF01112914 8503693

[22] van der Fits L Mourits S Voerman JS Kant M Boon L Laman JD Imiquimod-induced psoriasis-like skin inflammation in mice is mediated via the IL-23/IL-17 axis. J Immunol. (2009) 182:5836–45. 10.4049/jimmunol.0802999 19380832

[23] Nestle FO Conrad C Tun-Kyi A Homey B Gombert M Boyman O. Plasmacytoid predendritic cells initiate psoriasis through interferon-alpha production. J Exp Med. (2005) 202:135–43. 10.1084/jem.20050500 15998792 PMC2212894

[24] Finch AM Wong AK Paczkowski NJ Wadi SK Craik DJ Fairlie DP. Low-molecular-weight peptidic and cyclic antagonists of the receptor for the complement factor C5a. J Med Chem. (1999) 42:1965–74. 10.1021/jm9806594 10354404

[25] Yuan JS Reed A Chen F Stewart CN Jr. Statistical analysis of real-time PCR data. BMC Bioinform. (2006) 7:85. 10.1186/1471-2105-7-85 16504059 PMC1395339

[26] Gilliet M Boonstra A Paturel C Antonenko S Xu XL Trinchieri G. The development of murine plasmacytoid dendritic cell precursors is differentially regulated by FLT3-ligand and granulocyte/macrophage colony-stimulating factor. J Exp Med. (2002) 195:953–8. 10.1084/jem.20020045 11927638 PMC2193725

[27] Brawand P Fitzpatrick DR Greenfield BW Brasel K Maliszewski CR De Smedt T. Murine plasmacytoid pre-dendritic cells generated from Flt3 ligand-supplemented bone marrow cultures are immature APCs. J Immunol. (2002) 169:6711–9. 10.4049/jimmunol.169.12.6711 12471102

[28] Lindroos J Svensson L Norsgaard H Lovato P Moller K Hagedorn PH. IL-23-mediated epidermal hyperplasia is dependent on IL-6. J Invest Dermatol. (2011) 131:1110–8. 10.1038/jid.2010.432 21289639

[29] Hedrick MN Lonsdorf AS Shirakawa A-K Lee C-CR Liao F Singh SP. CCR6 is required for IL-23–induced psoriasis-like inflammation in mice. J Clin Invest. (2009) 119:2317–29. 10.1172/JCI37378 19662682 PMC2719919

[30] Di Cesare A Di Meglio P Nestle FO. The IL-23/Th17 axis in the immunopathogenesis of psoriasis. J Invest Dermatol. (2009) 129:1339–50. 10.1038/jid.2009.59 19322214

[31] Johansen C Usher PA Kjellerup RB Lundsgaard D Iversen L Kragballe K. Characterization of the interleukin-17 isoforms and receptors in lesional psoriatic skin. Br J Dermatol. (2009) 160:319–24. 10.1111/j.1365-2133.2008.08902.x 19016708

[32] Kollipara R Downing C Gordon R Tyring S. Interleukin-23 in the pathogenesis and treatment of psoriasis. Skin Ther Lett. (2015) 20:1–4. 25807335

[33] Chiricozzi A. Pathogenic role of IL-17 in psoriasis and psoriatic arthritis. Actas Dermosifiliogr. (2014) 105:9–20. 10.1016/S0001-7310(14)70014-6 25398488

[34] Lee E Trepicchio WL Oestreicher JL Pittman D Wang F Chamian F. Increased expression of interleukin 23 p19 and p40 in lesional skin of patients with psoriasis vulgaris. J Exp Med. (2004) 199:125–30. 10.1084/jem.20030451 14707118 PMC1887731

[35] Palamara F Meindl S Holcmann M Luhrs P Stingl G Sibilia M. Identification and characterization of pDC-Like cells in normal mouse skin and melanomas treated with imiquimod. J Immunol. (2004) 173:3051–61. 10.4049/jimmunol.173.5.3051 15322165

[36] Gibson SJ Lindh JM Riter TR Gleason RM Rogers LM Fuller AE. Plasmacytoid dendritic cells produce cytokines and mature in response to the TLR7 agonists, imiquimod and resiquimod. Cell Immunol. (2002) 218:74–86. 10.1016/S0008-8749(02)00517-8 12470615

