# Supplementary figures and images for: C5a/C5aR1 Pathway Is Critical for the Pathogenesis of Psoriasis
Source: Front Immunol. 2019 Aug 7;10:1866. doi: 10.3389/fimmu.2019.01866 (PMC6692472; doi:10.3389/fimmu.2019.01866)

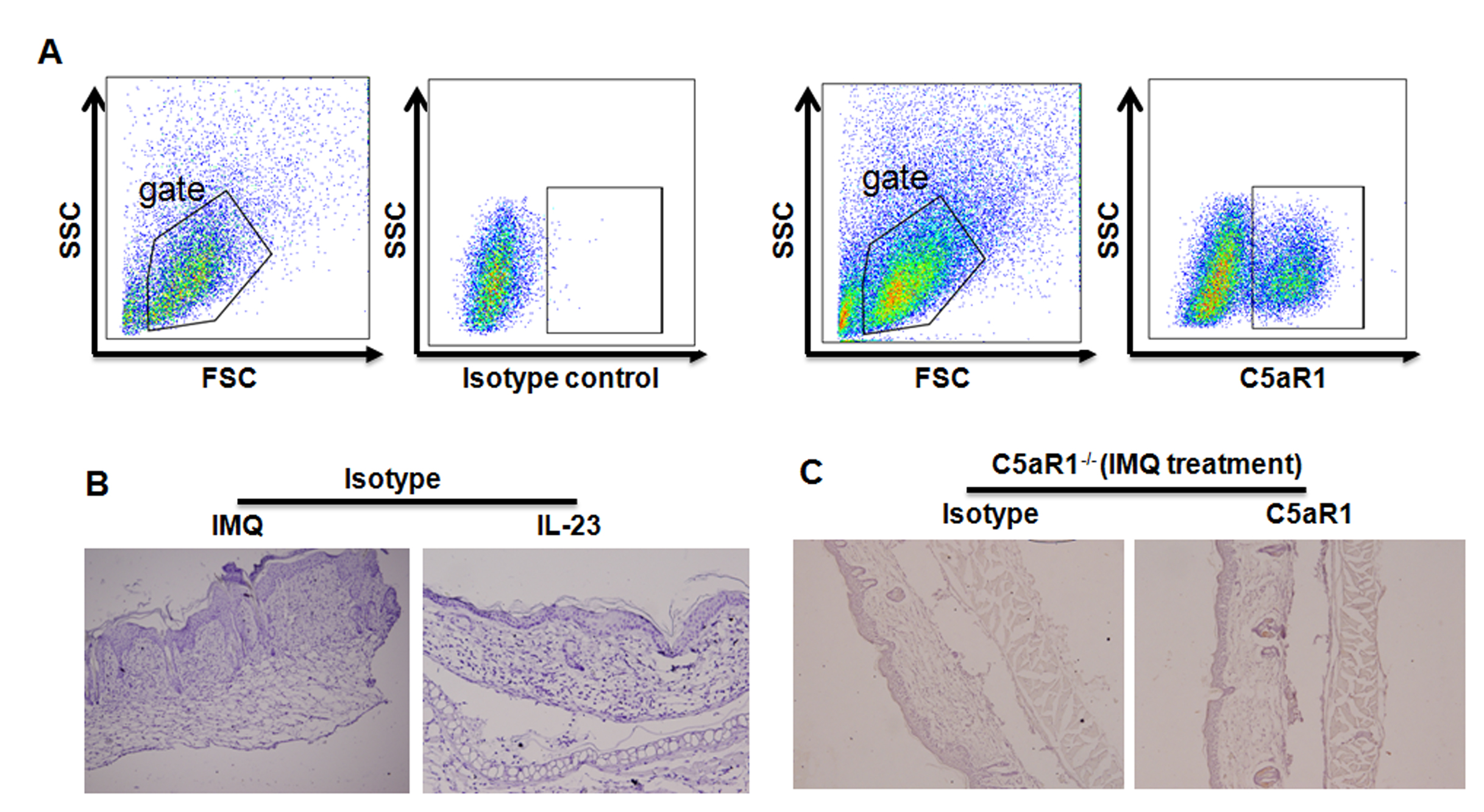

Supplement: Figure S1 — Isotype control and gating strategy of C5aR1 staining. After IMQ or IL-23 treatment, lesions skin sample of mice was collected and skin cells were isolated. (A) Skin single cell suspensions were stained with isotype IgG or anti-mouse C5aR1. The gating strategy is shown. (B) Skin paraffin sections were stained with rabbit IgG isotype antibody. (C) Skin paraffin sections from IMQ-treated C5aR1−/− were stained with rabbit isotype IgG or rabbit anti-mouse C5aR1 antibody. [file Image_1.JPEG]

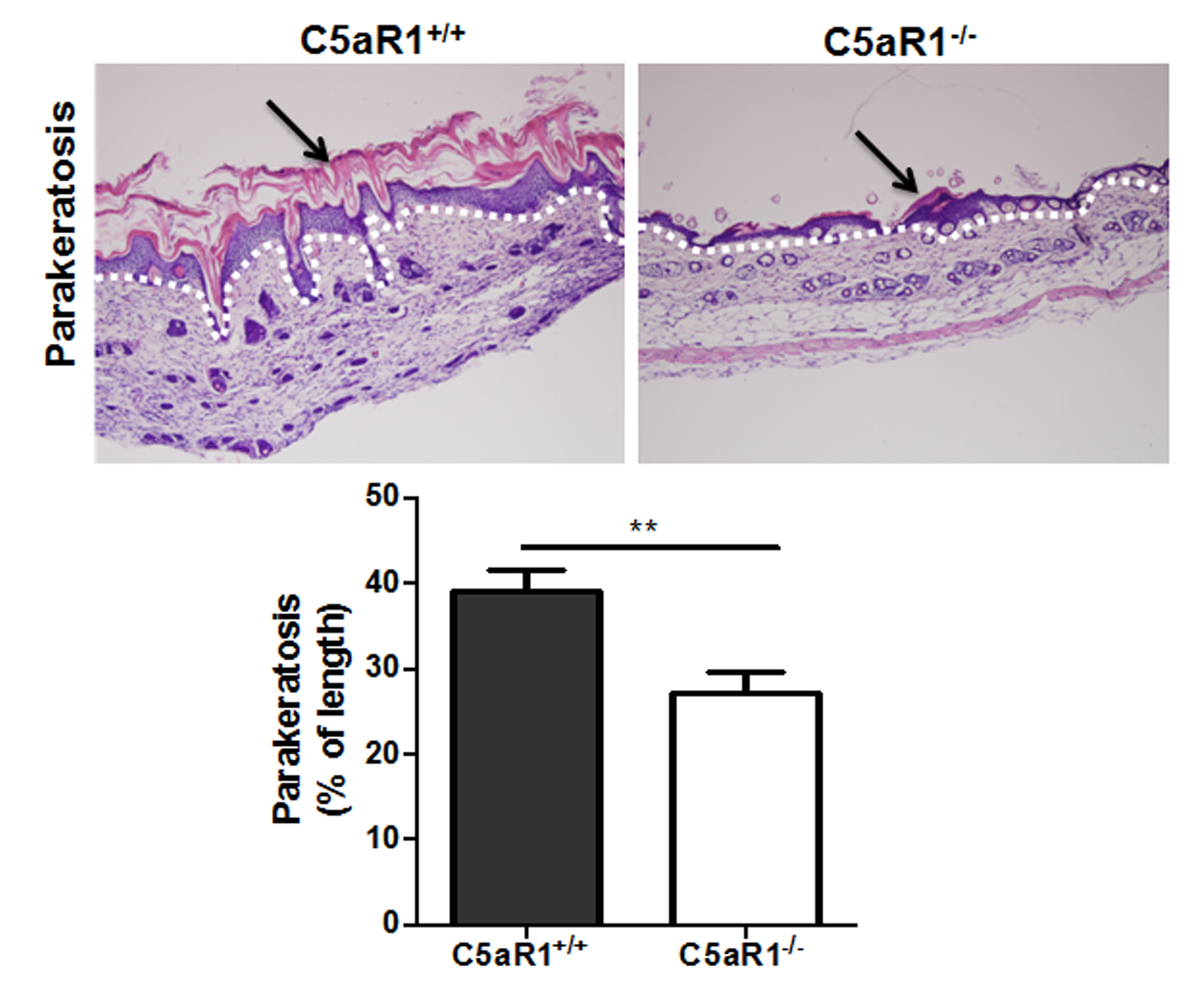

Supplement: Figure S2 — C5aR1 deficiency attenuated the parakeratosis of psoriatic skin in mice. The shaved back skin of C5aR1+/+ and C5aR1−/− mice were topically applied with IMQ for 6 days. H&E staining of mice skin sections and one representative image analysis for parakeratosis are shown. White dotted line: the location of the basal membrane. The length of parakeratosis was measured per high-power field (HPF, 200×) from 5 to 6 mice per group. Values are presented as mean ± SEM and data were obtained from at least two independent experiments. **p < 0.01. [file Image_2.JPEG]

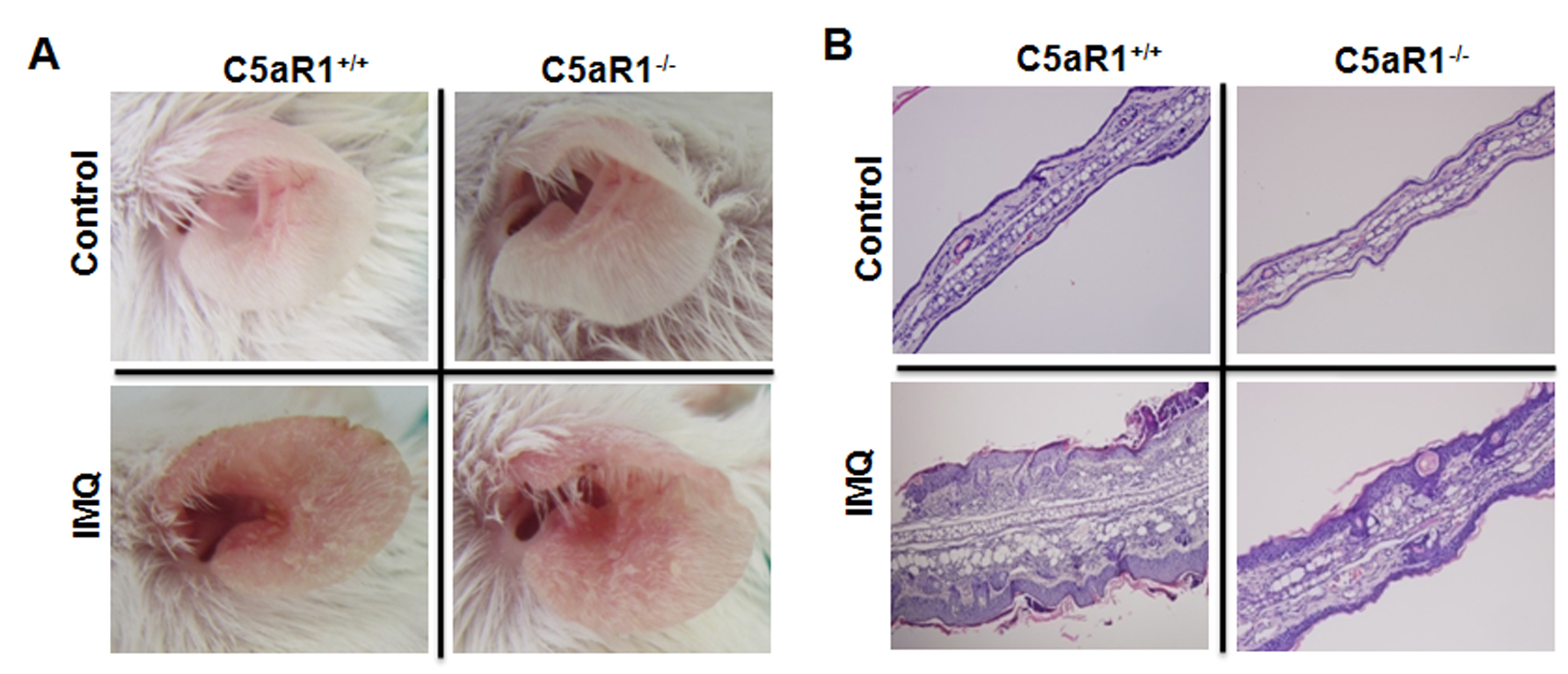

Supplement: Figure S3 — C5aR1 deficiency alleviated IMQ-induced psoriatic skin inflammation in ears. C5aR1+/+ and C5aR1−/− ears were topically treated with IMQ (Vaseline cream treatment as a control) for six consecutive days (n = 5–6 per group). Ear skin tissues were collected. (A) Macroscopic phenotype of psoriasiform lesions in C5aR1+/+ and C5aR1−/− mice. (B) H&E staining of ear skin sections. Data represent three independent experiments. [file Image_3.JPEG]

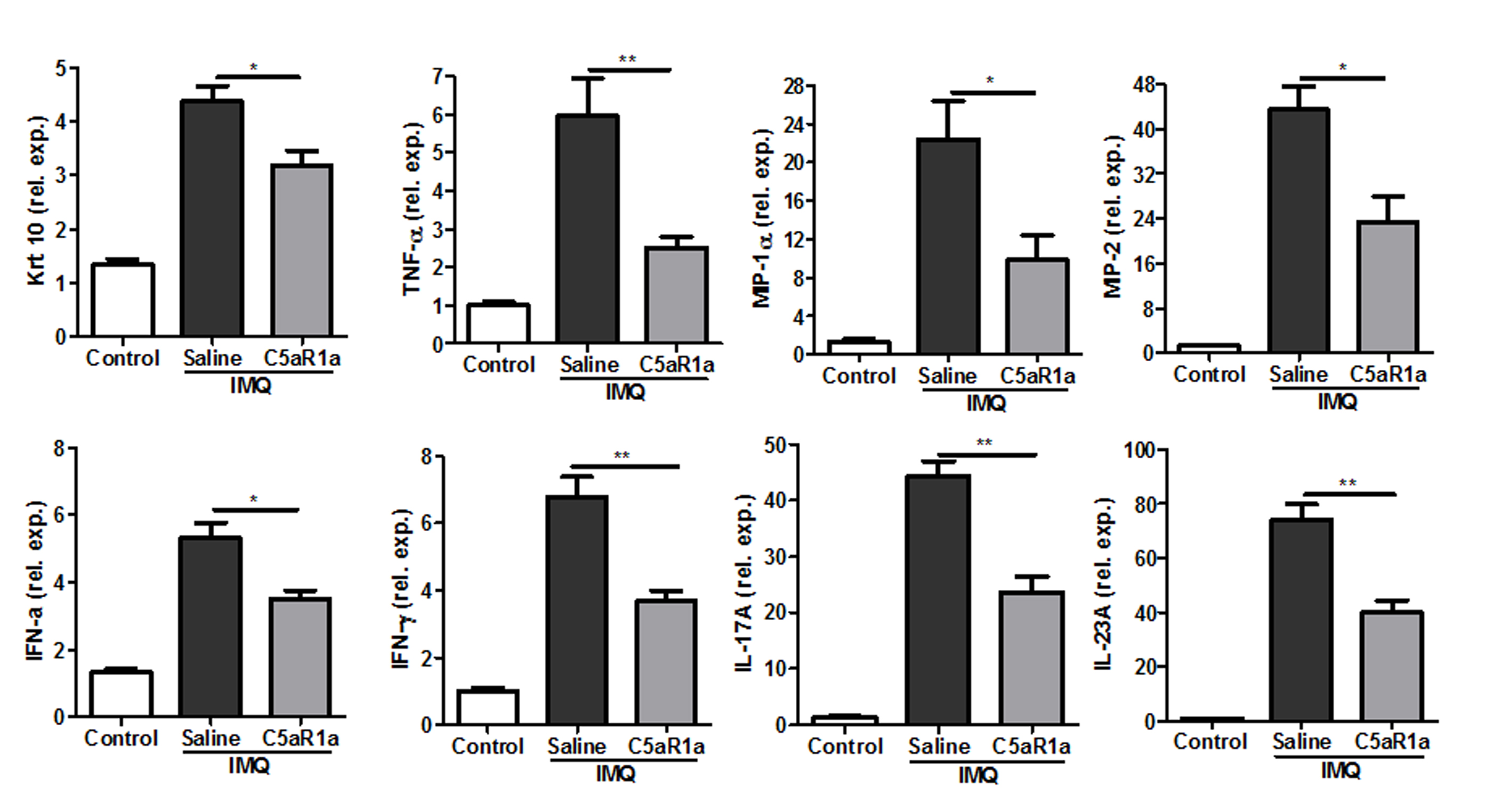

Supplement: Figure S4 — C5aR1 antagonist treatment led to a reduction in the levels of inflammatory response genes in psoriatic skin lesions. Mouse C5aR1 antagonist (1 mg/kg) or 0.9% saline was injected via intraperitoneal cavity 2 h prior to IMQ application in C5aR1+/+ wild type mice. After six consecutive days of IMQ application, skin samples were collected and expression of inflammatory response genes were analyzed by qRT-PCR (n = 4–8). Values are presented as mean ± SEM and data were obtained from at least three independent experiments. *p < 0.05; **p < 0.01. [file Image_4.JPEG]

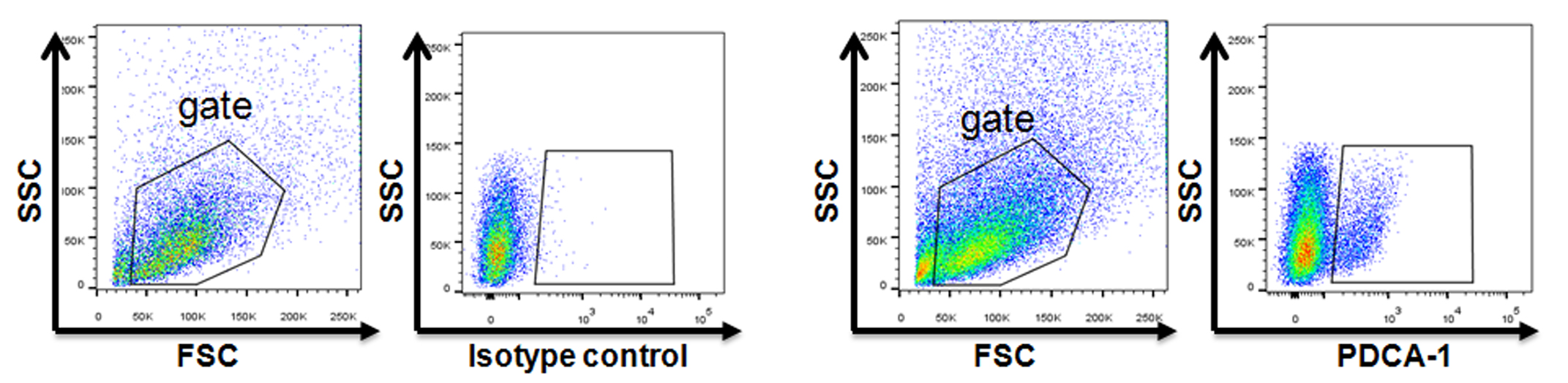

Supplement: Figure S5 — Gating strategy of pDCs staining. After IMQ treatment for indicated days, whole-skin cells were isolated from psoriatic skin lesions samples and stained with isotype IgG or anti-mouse PDCA-1 antibody. [file Image_5.JPEG]

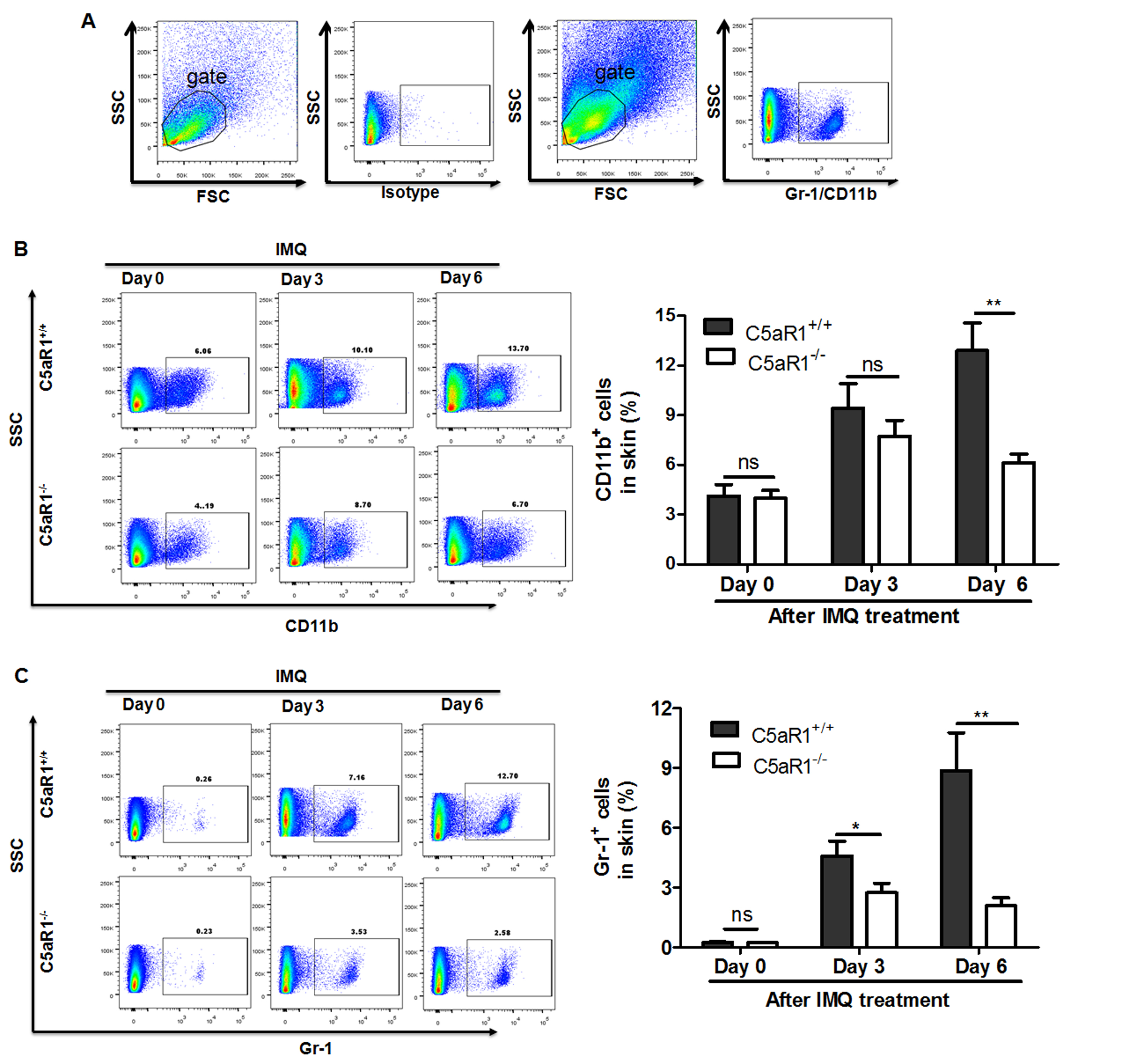

Supplement: Figure S6 — C5aR1 deficiency led to decrease in neutrophils and monocytes infiltration of psoriatic skin lesions in mice. C5aR1+/+ and C5aR1−/− mice were topically treated with IMQ for indicated days. Back skin samples were obtained. Infiltrated cells of skin lesions were isolated and the percentage of neutrophils and monocytes in the infiltrated cells was measured by FCM. (A) Gating strategy. (B) Monocytes (CD11b+ cells). (C) Neutrophils (Gr-1+ cells). (n = 5–6/group). Data were obtained from at least two independent experiments. ns, no significant, *p < 0.05; **p < 0.01. [file Image_6.JPEG]

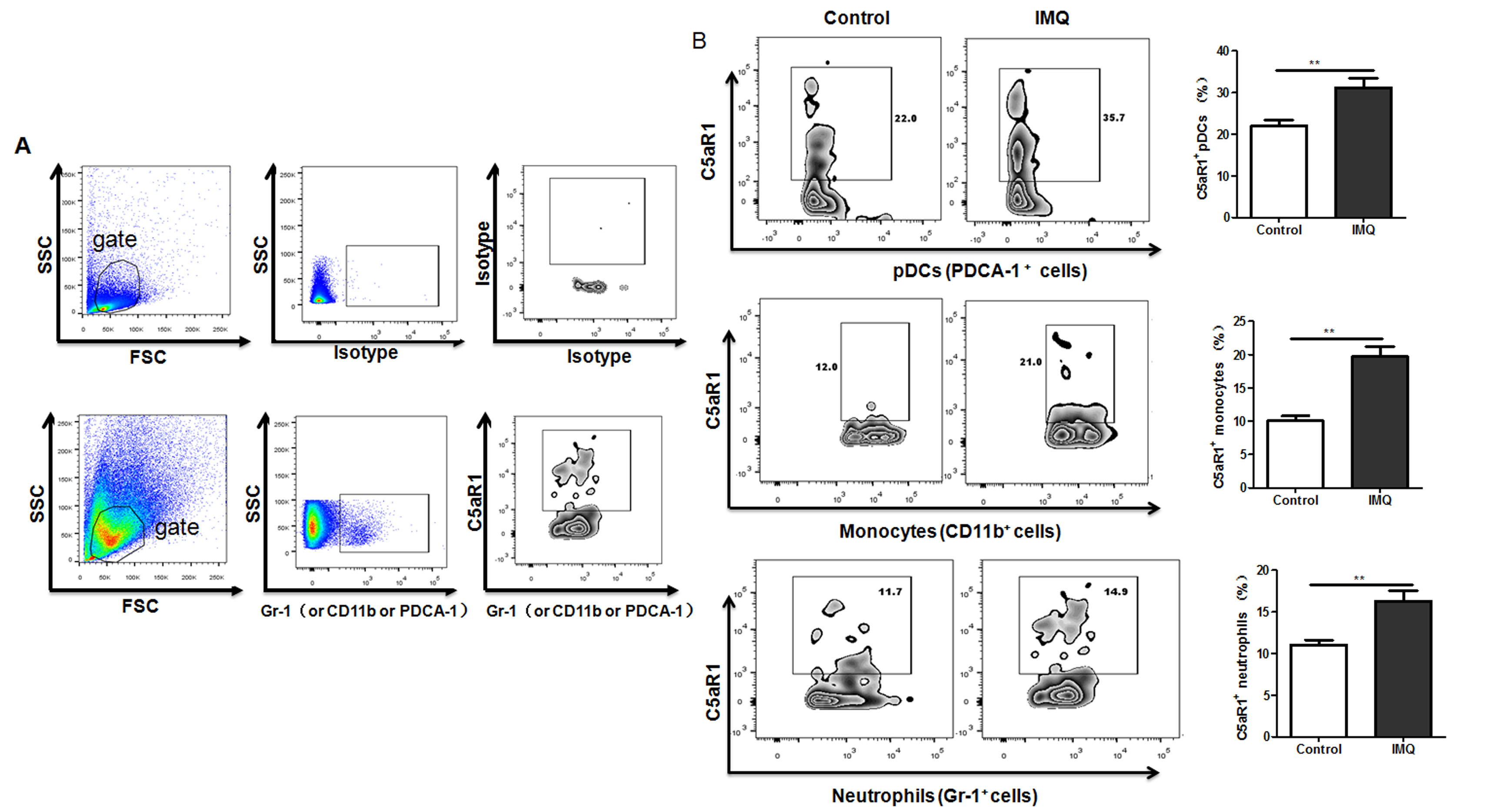

Supplement: Figure S7 — Increased C5aR1 expression in infiltrated skin cells in psoriatic skin lesions in mice. To induce a psoriatic animal model, IMQ was topically applied to the shaved back of wild-type (C5aR1+/+) mice for six days. Samples were collected and cells were isolated from the skin lesions. (A) Gating strategy. (B) C5aR1 expression on pDCs (PDCA-1+), monocytes (CD11b+) and neutrophils (Gr-1+) was assayed by FCM. (n = 5/group). ** p < 0.01. [file Image_7.JPEG]

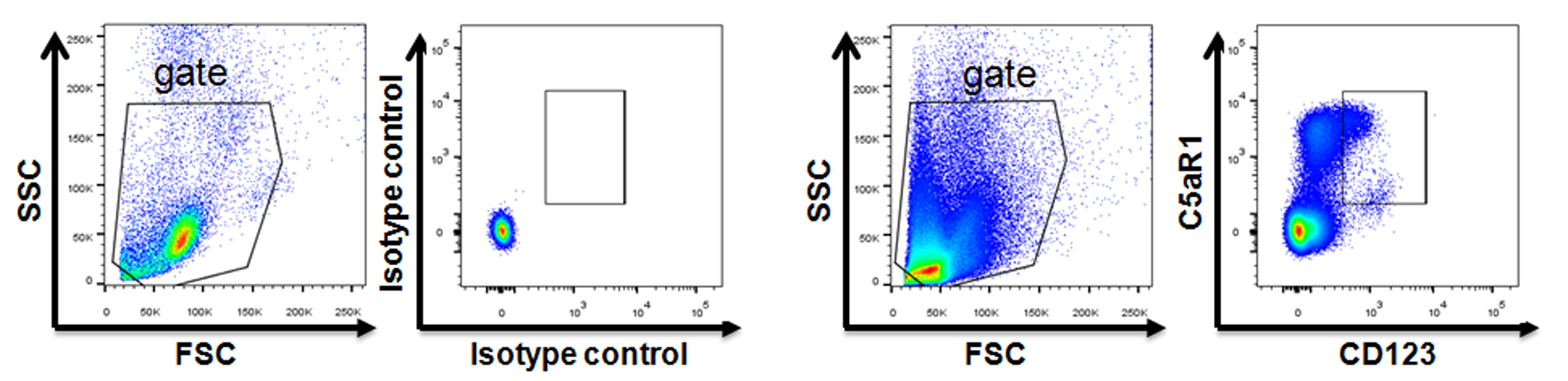

Supplement: Figure S8 — Gating strategy of CD123 and C5aR1 staining in human PBMCs. Human PBMCs were prepared as described in methods (n = 15/group), and then stained with isotype IgG or anti-human CD123 and C5aR1 antibodies, followed by flow cytometric analysis. [file Image_8.JPEG]

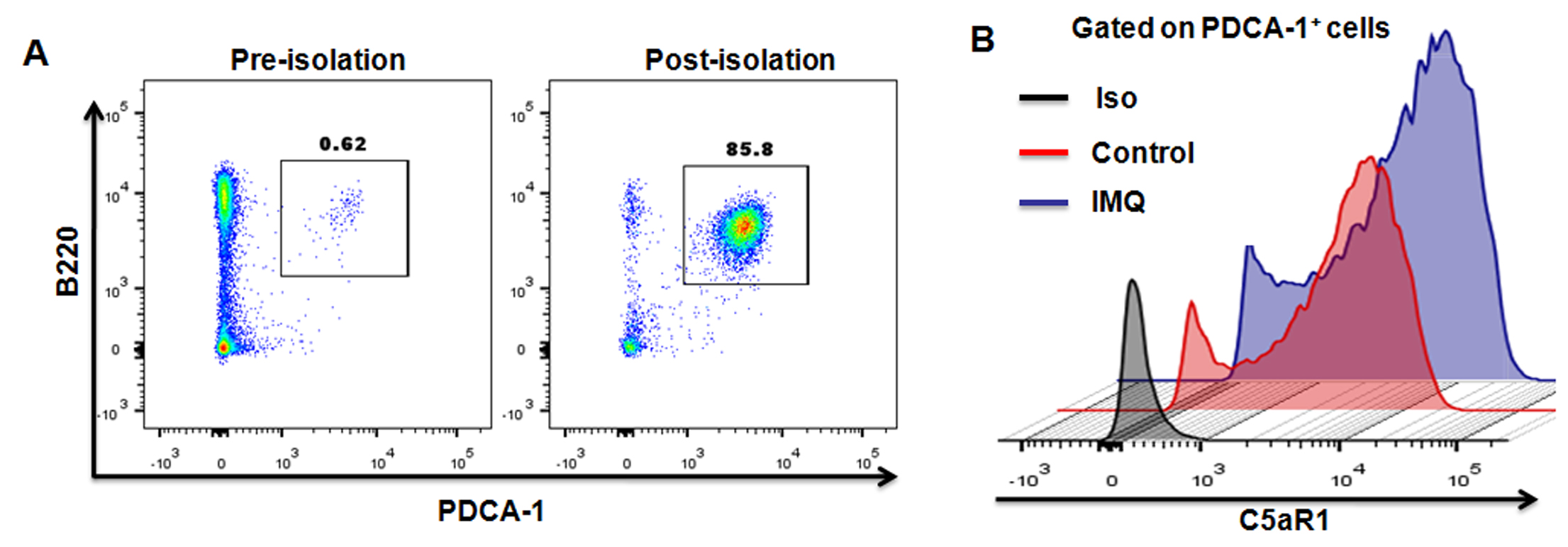

Supplement: Figure S9 — The purity of isolated pDCs and IMQ-induced C5aR1 expression of isolated pDCs. pDCs were isolated from splenocytes of C5aR1+/+ mice. (A) The purity of isolated pDCs (PDCA-1 positive cells) was measured by FCM. (B) C5aR1 expression on pDCs after IMQ stimulation for 24 h was measured by FCM. [file Image_9.JPEG]

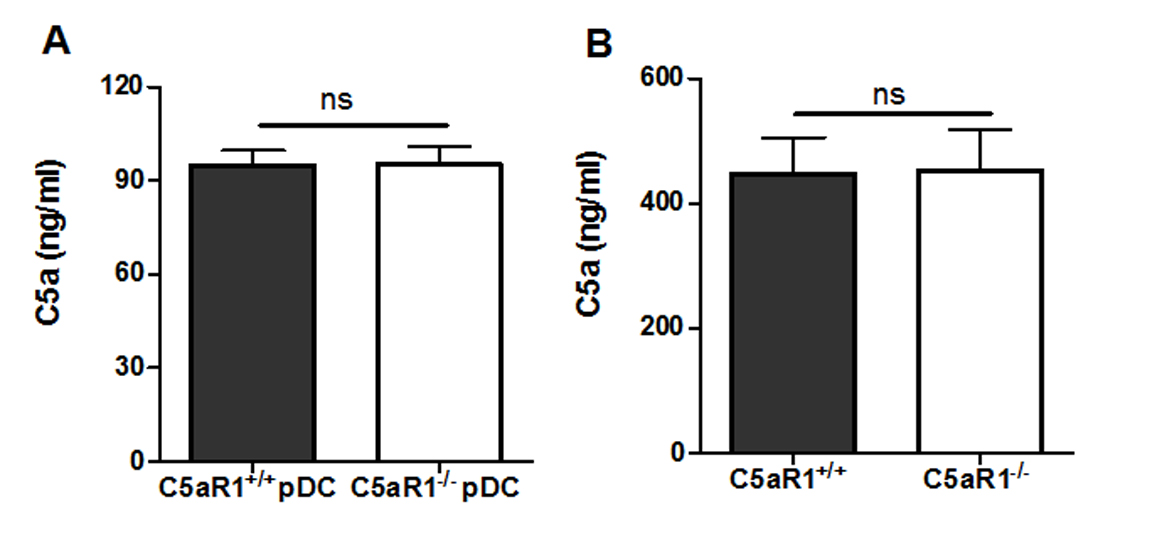

Supplement: Figure S10 — C5a levels in cultural supernatant of purified pDCs and in serum of naïve C5aR1+/+ or C5aR1−/− mice. (A) pDCs were isolated from splenocytes of C5aR1+/+ or C5aR1−/− mice and cultured in medium for 24 h. C5a levels in cell-free culture supernatant was measured by ELISA (n = 6/group). (B) C5a levels in serum from naïve C5aR1+/+ and C5aR1−/− mice measured by ELISA (n = 5/group). Values are presented as mean ± SEM and data were obtained from at least two independent experiments. [file Image_10.JPEG]
